# Unobtrusive Health Monitoring in Private Spaces: The Smart Vehicle

**DOI:** 10.3390/s20092442

**Published:** 2020-04-25

**Authors:** Ju Wang, Joana M. Warnecke, Mostafa Haghi, Thomas M. Deserno

**Affiliations:** Peter L. Reichertz Institute for Medical Informatics of TU Braunschweig and Hannover Medical School, D-38106 Braunschweig, Lower Saxony, Germany; joana.warnecke@plri.de (J.M.W.); mostafa.haghi@plri.de (M.H.); thomas.deserno@plri.de (T.M.D.)

**Keywords:** digital health, sensor, smart vehicle, health monitoring

## Abstract

Unobtrusive in-vehicle health monitoring has the potential to use the driving time to perform regular medical check-ups. This work intends to provide a guide to currently proposed sensor systems for in-vehicle monitoring and to answer, in particular, the questions: (1) Which sensors are suitable for in-vehicle data collection? (2) Where should the sensors be placed? (3) Which biosignals or vital signs can be monitored in the vehicle? (4) Which purposes can be supported with the health data? We reviewed retrospective literature systematically and summarized the up-to-date research on leveraging sensor technology for unobtrusive in-vehicle health monitoring. PubMed, IEEE Xplore, and Scopus delivered 959 articles. We firstly screened titles and abstracts for relevance. Thereafter, we assessed the entire articles. Finally, 46 papers were included and analyzed. A guide is provided to the currently proposed sensor systems. Through this guide, potential sensor information can be derived from the biomedical data needed for respective purposes. The suggested locations for the corresponding sensors are also linked. Fifteen types of sensors were found. Driver-centered locations, such as steering wheel, car seat, and windscreen, are frequently used for mounting unobtrusive sensors, through which some typical biosignals like heart rate and respiration rate are measured. To date, most research focuses on sensor technology development, and most application-driven research aims at driving safety. Health-oriented research on the medical use of sensor-derived physiological parameters is still of interest.

## 1. Introduction

Unobtrusive and continuous health monitoring in private spaces uses sensor technology without introducing any inconveniences to the patient’s normal life [1]. With respect to “private spaces”, we refer to a living environment with limited public access, such as a home, apartment, or a privately owned car [2]. In sensor-enhanced private spaces, health-related information is collected continuously and critical changes or events are captured automatically. Furthermore, the collected information reflects the natural reality and promising services, including emergency detection, disease management, and health status feedback, and advice becomes achievable [3].

The Internet of Things (IoT) facilitates the ubiquitous sensing of all aspects of people’s lives, including health, entertainment, activities, and communication [4]. Big data (variety, velocity, volume) is collected unobtrusively and artificial intelligence (AI) is applied to early detect diseases or predict health status [5,6]. As of today, people’s private spaces are equipped with advanced technology, which is reshaping their lifestyles. For example, smart wearables track activities. AI-based personal assistants, such as Amazon Alexa or Google Home, have not only changed human-machine interaction but established these technologies as part of regular life [7], which also includes, for instance, autonomous driving.

Personal mobility is a distinctive trait of modernity. In many countries, people spend a considerable amount of time in cars: the average daily time is about 1 and 1.1 h in the United States [8] and in Germany [9], respectively. Furthermore, the private vehicle is one of the best-equipped environments in our daily life. This provides a great opportunity to convert the vehicle into a health monitoring facility [2] and to use the time people spent in their cars for health monitoring [10,11].

For many years, in-vehicle health monitoring has been the focus of research. This includes environmental, physiological, and behavioral monitoring:

- *Environmental parameters* include temperature, air quality, humidity, weather and light conditions, and speed. They are captured already by default for in-car well-being and driver’s assistance systems.
- *Physiological parameters* typically include vital signs; in particular, heart rate (HR), respiration rate (RR), body surface temperature, and skin impedance. More advanced parameters can be measured with special sensing devices.
- *Behavioral parameters* quantify physical activities during the drive to reflect the driver’s attention level, tiredness, and well-being.

With respect to physiological parameters, Naziyok et al. reviewed contact-less monitoring for general wards and highlighted ballistocardiography (BCG), radar, and thermography for HR, RR, and cardiopulmonary signals, respectively [12]. More recently, Leonhardt et al. comprehensively reviewed unobtrusive vital-sign monitoring in automotive environments [10]. Based on cardio-respiratory and thermo-regulatory couplings, they obtain bio-electrical, mechanical, and thermal effects. Sensors, such as electrocardiography (ECG), capacitive ECG (cECG), radar, BCG and seismocardiography (SCG), video imaging, photoplethysmography (PPG) and PPG imaging (PPGI), magnetic induction (MI), and thermography capture body surface potentials, displacements and temperatures, the superficial perfusion, and the intrathoracic impedance. Bruser et al. particularly focus on cardio-respiratory parameters [13].

The use of camera sensors allows not only to directly observe the driver’s activities [14] but also delivers vital signs. For instance, oxygen saturation is measured by attaching a light-emitting diode (LED) and photo-diode to the steering wheel [15] as well as from analyzing images captured by a camera attached to the windscreen or the control panel dashboard [16]. The RR can be measured when deploying a piezoelectric sensor or an accelerometer on the seat belt [11,15] but also from radar signal [17].

To date, several authors use physiological information to monitor the driver state, detect fatigue, or assess stress, and the data is fed into driving assistance systems [18,19,20]. These applications require robustness during the entire drive, which is hardly reached.

Medical applications, in contrast, profit from the regularity of every-day use and cope with dropouts during the ride. However, existing research does not provide a guide to currently proposed sensor systems for in-vehicle monitoring. In particular, the following questions need to be answered.

1. Which sensors are suitable for in-vehicle data collection?
2. Where should the sensors be placed?
3. Which biosignals or vital signs can be monitored in the vehicle?
4. Which purposes can be supported with the health data?

## 2. Methods

In this paper, we focus on unobtrusive continuous health monitoring in a smart vehicle, which we consider a private environment or a private space. We present terminology for sensors, locations, biosignals, and purposes and applied a comprehensive literature review to answer the stated questions.

### 2.1. Terminology of Unobtrusive In-Vehicle Health Monitoring

The primary goal of driving is to reach a predefined destination. The driver perceives information about the environment, such as weather, road conditions, and traffic signs, and accordingly controls the vehicle [21]. Driving consist of tasks on different levels [22]:

- Strategic tasks (e.g., choice of route);
- Navigational tasks (e.g., adherence to the chosen route);
- Traffic-related tasks (e.g., interacting with other road users);
- Adherence to rules (e.g., traffic signs and signals);
- Tasks related to the road (e.g., chosen position within traffic); and
- Speed control (e.g., maintenance of the speed according to road situation).

**Figure 1:**
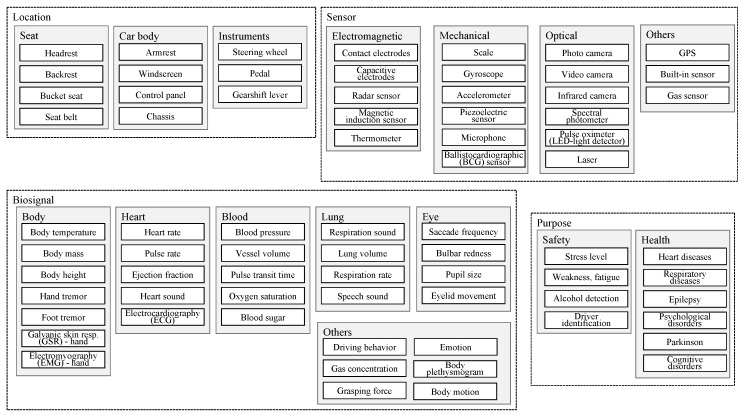
Terminology of unobtrusive in-vehicle health monitoring.

For these tasks, objects such as steering wheel, windscreen, mirrors, pedals, or the dashboard (speedometer) are used, and their context, location have to be considered [23,24]. Accordingly, we propose a flat terminology that covers unobtrusive sensors (electromagnetic, mechanic, optic), their locations (seat, chassis, instruments), and the biosignals or vital signs (body, heart, blood, lung, eye) that can be obtained by the sensors and the purposes (Figure 1).

### 2.2. Literature Retrieval

Using our terminology of unobtrusive in-vehicle health monitoring (Figure 1), we developed the search string that reflects two aspects:

- *Biosignal* consists of general terms, such as biosignal, biological signal, physiological signal, physiological parameter, vital signal, vital sign, vital parameter, and commonly seen specific biosignals terms, such as ECG, electrocardiograph, heart rate, heart rate variability, heartbeat, respiration rate, breathing rate, breathing, body movements;
- *Vehicle* consists of terms regarding the vehicle, such as car, vehicle, automobile, automotive, drive, driving, driver.

We connect the terms within and across each aspect with logic or and and, respectively (Appendix A). We applied the search string to the title of articles from three databases: PubMed, IEEE Xplore, and Scopus on 16 December 2019. To reflect only up-to-date research, the publishing date spans the last decade (2009–2019). Furthermore, we restricted responses to the English language. After we combined all returned records, we removed duplicated papers. Then, we screened titles and abstracts according to Section 2.3 and excluded irrelevant records. Afterward, we studied the full papers and excluded work that was published already in included articles (in such cases, we found a large overlap in the content of papers of the same authors).

### 2.3. Review Criteria

We reviewed the retrieved full papers to identify research focusing on the application of sensor technology for unobtrusive health monitoring in the vehicle. As several persons performed the two-stage review, we defined the following criteria:

- Inclusion
  – Unobtrusive sensors are part of the method;
  – The sensors are used to collect heath-relevant data, i.e., behavioral or physiological parameters.
- Exclusion
  – The sensors are body-attached, wearable, or implanted;
  – Sensor data is not used for biosignal or health state monitoring;
  – Research is not on humans.

When analyzing the full texts, we extracted the type of sensor and its location, the targeted biosignals or medical parameters, and the main purpose of the research. Possible options for the purposes are:

- *Sensor development* for measuring a certain health parameter;
- *Application* of sensor data for health (i.e., disease management, diagnostics, prediction) or safety (Figure 1).

With respect to the conditions of an experiment, possible options are:

- *On-road driving*: the experiment was performed with naturalistic driving, where the subjects were required to drive a car on real roads;
- *Driving simulator*: the subjects were required to simulate driving activities on a driving simulator;
- *Laboratory setting*: a driving-like setting up or a separated (part of a) vehicle was equipped with sensors (e.g., seat, steering wheel), but no driving activity was simulated.

We further tracked the number of subjects that participated in the experiment.

## 3. Results

**Figure 2:**
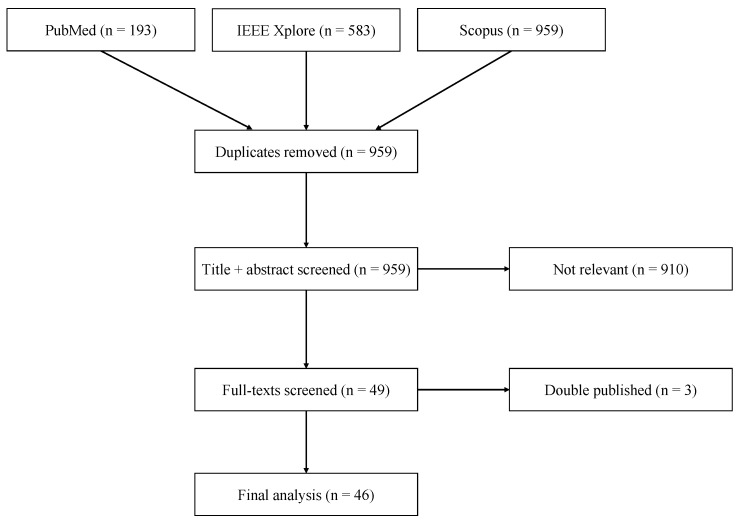
Result of literature review.

The initial search query on PubMed, IEEE Xplore, and Scopus resulted in 959 records after removing duplicates, of which 49 papers remained after assessing titles and abstracts (Figure 2). When reviewing the full texts, we excluded three papers additionally, due to a high overlap in content to already included papers of the same authors (double publishing). Finally, 46 papers remained for text analysis (Table 1).

**Figure 3:**
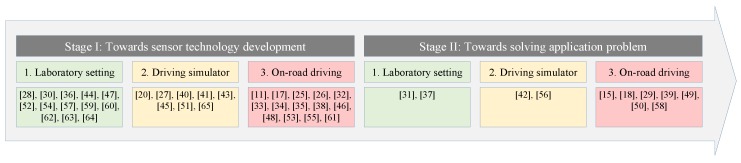
Stages of current research. The bottom row lists reference numbers of the papers.

Based on the review criteria (i) sensor development vs. application and (ii) laboratory setting vs. diving simulator vs. on-road driving, we categorized all papers into six groups (Figure 3).

The majority of research (n=35) focuses on the development of sensor technology. Only a few works (n=11) are toward problem-solving. Fourteen of the 35 papers for sensor development have conducted on-road driving tests, but most of them with only a few subjects (1–5). Exceptions are the work of Kuo et al. [25] and Lee et al. [26] with ten subjects in an on-road drive test. Nine out of 11 application-oriented research focus on safety, and only two papers on health-related issues.

**Figure 4:**
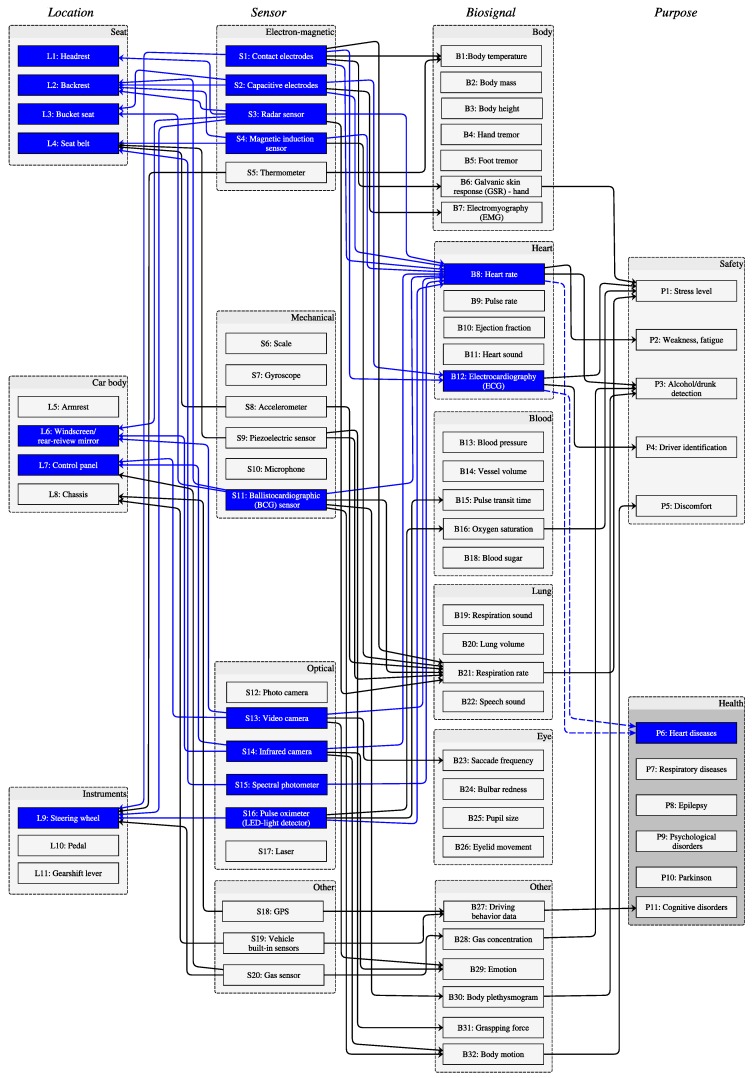
In-vehicle health monitoring: state-of-the-art. We code the nodes in columns Location, Sensor, Biosignal, and Purpose with L[n], S[n], B[n], and P[n], respectively, and all interconnections with their supporting nodes. For instance, S2—L3 represents the reference where the authors use a capacitive electrode (S2) embedded in the bucket seat (L3) (Table 2).

According to our terminology (Figure 1), we depict the state-of-the-art for in-vehicle health monitoring in a graph that links columns for location, sensor, biosignal, and purpose (Figure 4). The interconnections are based on all 46 papers. In other words, we draw interconnections only on evidence in the literature. Also, we labeled the interconnections with the reference numbers of the corresponding papers. To enhance readability, we collected all interconnections and their supporting literature separately in Table 2.

### 3.1. Sensors

In total, 15 types of sensors were found in the included papers. Contact (dry) electrode, capacitive electrode, radar sensor, and video camera are the most popular sensing devices, which can be found in 11, 9, 8, and 7 papers, respectively. The papers involving the contact or capacitive electrodes were mostly published from 2009 to 2014 (n=18), while the video-related work was mainly published from 2014 to 2018 (n=8). Several authors collect ECG, EMG, or galvanic skin response (GSR) through electrode and corresponding amplifiers and filters [15,20,27,29,30,37]. When cameras are in use, the authors target PPG-derived HR or heart rate variability (HRV) [25,44,46,53,61]. Some work uses cameras naturally for activity or emotion monitoring [28,43]. The pulse oximeters are attached to the steering wheel and measure HR or RR [15,27,66]. BCG sensors are widely explored for in-vehicle scenarios. HR and RR are the typical health parameters that can be extracted from BCG [28,31,57]. In recent years, a radar sensor has become a device for recording physiological parameters, such as HR and RR [45,48,55,62,64]. Furthermore, two teams use vehicle-built-in sensors, such as GPS, to assess the driving behavior or the driver’s mental health [49,58].

### 3.2. Locations

All included research assumed that the driver was the monitored person, i.e., driver-centered. Although there are plenty of options for human-vehicle interaction, only a limited number of locations are actually used for health monitoring. Car seats (n=20) and steering wheels (n=18) most frequently host sensing devices. The car seat (backrest and seating area) is a suitable location for capacitive electrodes [20], BCG sensors [31,57], magnetic induction sensors [33,51] and radar sensors [45,48]. Since the hands are the unique body part of the driver that directly contacts the vehicle, the steering wheel is equipped with contact electrodes, a pulse oximeter, and a thermometer [27,28,30,66]. The control panel, windscreen, and the windscreen-mounted rear-mirror mostly host a camera [25,53,56,61] or a gas sensor [50]. A modern vehicle is equipped already with many sensors for drive-train control, safety, and comfort [67]. The data from these built-in sensors are potential resources for behavior monitoring, and current research considers respective data interfaces (on-board diagnostics) [49,58].

### 3.3. Biosignals

HR (n=24) and RR (n=15) are the most frequently measured health parameters. HR data is often measured indirectly based on ECG, BCG, remote PPG (rPPG), or radar signals [18,31,68]. Jung et al. derive the HRV from the HR data [18]. RR is usually measured via PPG and BCG [15,57]. Some research uses magnetic and radar sensors [51,68]. Aiming at identifying the driver’s stress level [15,27], four papers covered GSR signals [15,27,28,30]. Some research collects data on body movement and facial emotion by video or infrared cameras [43,56]. Using such data, the authors detected abnormal situations such as discomfort and drowsiness. The driving behavior may also potentially indicate mental health problems, such as cognitive ability, particularly for old people. Built-in sensors can provide the number of trips, driving duration and distance, time driving on local streets and highways, time driving during daylight, after dusk, or at night, and the number of hard breaks or accelerations [58]. The GPS device records environmental parameters like the driving areas [49].

### 3.4. Purposes

We found eleven papers toward solving health problems. However, most of them (n=9) aim at improving driving safety. Stress and driving fatigue are detectable through HR and HRV [15,18,29,39]. EMG may also be incorporated as a source to extract features [42]. In order to detect drunk driving, the alcohol concentration is measured through a gas sensor [50], and driving behaviors under drunk conditions also could be a clue [31]. Several works explore the effectiveness of ECG for driver identification [37]. Discomfort detection benefits from monitoring body motion by an infrared camera and pressure pad [56].

Despite the popularity of research on developing ECG detection and HR measurement, we found no research using the measured data for solving clinical problems. Only two papers conducted strict subject selection (elderly people) and were aimed at Alzheimer’s disease [49,58].

## 4. Discussion

In this paper, we analyzed up-to-date research on in-vehicle health monitoring using unobtrusive sensors, their locations, the biosignals recorded, and the (medical) purposes of recording. Based on our in-vehicle health monitoring terminology, we systematically reviewed the literature. Three databases performed our query and return almost a thousand responses. Interestingly, the records returned from PubMed and IEEE Xplore are entirely subsets of those from Scopus. This is in contrast to our findings in previously performed systematic reviews [69] and may be explained with the special focus on vehicles, which might not be covered completely in PubMed, as vital signs and medical applications are not in the focus of IEEE Xplore. However, the high sensitivity of our retrieval strategy is indicated by the fact that only less than 5% of the initially returned records survived our taxonomy-based standardized selection process and was included in this report (46/959=4,8%).

We developed a graph (Figure 4) that is based on our terminology (Figure 1) and our review results. It can serve as a framework or guide when developing in-vehicle health monitoring. A list of possible sensors as well as their suggested locations can be derived from the biomedical data that is needed for the respective purpose or application. Assuming the driver’s heart parameters, such as HR, HRV, and RR, can be measured reliably while driving, a medical check-up could be done in the car on a daily basis during commuting. ECG- or BCG-based monitoring of these parameters enables early detection of heart disorders, such as atrial fibrillation [70,71], the precursor of stroke. To this end, the possible routes are highlighted with blue lines in Figure 4. Furthermore, we list the health purposes that can be supported by in-vehicle health monitoring, including heart diseases, respiratory diseases, epilepsy, psychological disorders, Parkinson’s, and cognitive disorders. In addition, the annotated connections with references (Table 2) denote the weight of the connections. The connections with more supporting references suggest the promising approaches with higher feasibility. The table also provides clear linkages to the related references.

The graph in Figure 4 provides the key to answer our initial questions (Section 1):

- *Which sensors are suitable for in-vehicle data collection?* Contact and capacitive electrodes capture ECG, and HR and HRV are computed from the recordings. Radar and magnetic induction sensors also are used for HR and RR measurements by detecting electron-magnetic signals due to organ movements, while BCG and piezoelectric sensors as well as accelerometers achieve similar goals through mechanical changes. Cameras provide video data, from which rPPG is generated. Furthermore, HR or HRV is extracted from the video data. Other work profiles driving behavior from vehicle built-in sensors in combination with GPS.
- *Where should the sensors be placed?* Car seat and steering wheel host sensors that are in direct or indirect contact to the driver, e.g., capacitive and contact electrodes, respectively. The control panel and the windscreen are the suitable locations to mount video or infrared cameras.
- *Which biosignals or vital signs can be monitored in the vehicle?* A variety of biosignals or vital signs are monitored already in the vehicle, including body-related (e.g., body temperature, EMG, GSR), heart-related (e.g., HR, ECG), blood-related (e.g., pulse transit time, oxygen saturation), lung-related (e.g., RR), and eye-related (e.g., saccade frequency) parameters. Some work focus on other information like driving behavior, gas concentration, emotion, body plethysmogram, grasping force, and body motion.
- *Which purposes can be supported with the health data?* Driving requires intensive engagement in terms of both mental and physical efforts. The performance of driving is also associated with health problems, such as cognitive disorder [58,72]. As known, essential tremor is associated with incident dementia [73], and the monitoring of hand/foot tremor, for example, by detecting the operation of steering wheel or pedal gives us the possibility to assess the driving performance. The research in Stage I (Figure 3) points out the hidden clinical values of measuring biosignals while driving. However, most application-driven research (Stage II in Figure 3) is aimed at driving safety, such as fatigue detection and drunk driving. Though in-vehicle health assessment has potential in monitoring cognitive disorders, it is not yet developed to deliver medical monitoring in a clinical sense.

So far, a standardized terminology for in-vehicle health monitoring has not been established. For example, *control panel* is also called *dashboard* in many cases; GSR is also known as electrodermal activity (EDA). The proposed terminology (Figure 1) in this work, on the one hand, may promote the consistency in scientific communication and, on the other hand, it can form a structured description of a monitoring system. Surely the scientific community needs to further develop the terminology.

Driving makes the vehicle a dynamic environment. To improve the robustness of health-monitoring systems, one biosignal or vital sign can be measured by multiple sensors. For example, HR is obtained from ECG and PPG. Thus, multiple sensors yield a recording system with redundancy. Furthermore, when multiple parameters are intended to be observed, a body area network (BAN) is formed [74]. As a result, sensor coupling is an issue that must be considered for signal and data synchronization in future work.

Besides sensors, the vehicle is equipped with actuators [67]. Coupling such actors with health monitoring can perform signaling alarms in emergency cases, for example, when detecting heart attacks. Sensors and actuators communicate via the in-vehicle information system (IVIS) [75]. Schneider et al. transferred the capacitive ECG through the controller area network (CAN) bus [38]. Open issues include, but are not limited to, (1) how much of the large amount of continuously monitored data should be stored within the IVIS, (2) how the health data shall be managed, (3) how the data fusion workflow shall be implemented in the IVIS, and (4) data exchange between IVIS and health information systems (HIS).

The existing literature has shown the potential of unobtrusively monitoring health data in a mobile environment. The IoT sensors are nevertheless only able to measure physiological states but not interpret the underlying causes of them. Therefore, intrinsically linked mechanisms need to be put in place so that the collected data can be analyzed and interpreted by health professionals.

The reviewed literature focuses on health monitoring of the driver while passengers are rarely considered. We also presume that drivers will play a key role as their interactions with the vehicle provides relevant information. If we consider multiple occupants, the seating location provides a key to assign the data to the subject. However, it is a question of in-vehicle data storage and processing whether passengers shall be monitored at all. This will require instantaneous cloud transfer of data and cloud-based identity management. Then, scenarios can be satisfied where, for instance, a person sits in the driver seat and then moves to the passenger seat of a different car.

Digital technologies introduce ethical issues such as privacy vulnerabilities for users [76]. Accompanied with the vehicle telematics, physiological information tells far beyond the driving behavior. The driver can be identified with the physiological data [37]. The ownership and access to the data set are still open. Avoiding health information disclosure must be considered from the very beginning [77], and any link between the vehicle and external systems must be secured to protect from hacking health data.

To foster the car as a diagnostic space, strictly designed clinical studies need to be conducted. So far, the number of subjects is still low, and the significance of the effects of monitored data has not shown up. Semantic integration of sensor data for in-vehicle health management requires the establishment of standards [78].

## 5. Conclusions

As an equipped private space, the smart vehicle is a promising facility for health monitoring. This work provides a guide with regard to sensors, location, biosignals, and the purpose of currently proposed sensor systems for in-vehicle monitoring. Potential sensors can be derived from the biomedical data that is needed for the respective purpose or application. The suggested locations for the corresponding sensors are also linked. The annotated connections with references denote the weight of the connections and provide clear linkages to the related references. To the four sub-questions: (1) Unobtrusive sensors, which are based on electro-magnetical, mechanical, and optical mechanisms, are used for in-vehicle health data collection. (2) Driver-centered locations, such as steering wheel, car seat, and windscreen, are the most commonly used locations to host the sensors. (3) Typical physiological signals/parameters, such as ECG, HR, RR, body temperature, can be measured reliably, even while driving. (4) To date, most research has focused on sensor technology development. Research on mental health assessment and profiling the driving behavior is on track. However, health-oriented research on the medical use of physiological parameters is still on-demand. Furthermore, the terminology used in literature analysis may promote consistency in scientific communication and form a structured description of a monitoring system.

## Figures and Tables

**Table 1:** Included research on in-vehicle health monitoring. The articles are ordered by ascending year. NA: the information is not available.

No.	Ref	Year	Sensor	Location	Biosignal/Parameter	Objective	Test Setting	# of Subjects
1	[15]	2009	Contact electrode, pulse oximeter, capacitive electrode (conductive textile), piezoelectric sensor	Steering wheel, bucket seat, seat belt	GSR, PPG, ECG, RR	Safety: driver’s stress	On-road driving	4
2	[27]	2010	Pulse oximeter, contact electrodes	Steering wheel	PPG, GSR	Sensor development	Driving simulator	24
3	[28]	2010	Contact electrode (IDAT microsensors, PGR and ECG sensors), BCG sensor (pressure)	Steering wheel, bucket seat	GSR, body temperature, HR, ECG, respiration rate	Sensor development	Laboratory setting	NA
4	[29]	2010	Contact electrode (conductive fabric), pulse oximeter	Steering wheel	ECG, PPG → HR, HRV	Safety: drowsiness evaluation	On-road driving	2
5	[30]	2010	Contact electrode, thermometer (infrared), pulse oximeter, capacitive electrode	Steering wheel, seat backrest	ECG, GSR, PPG, temperature (finger)	Sensor development	Laboratory setting	NA
6	[31]	2011	BCG sensor (air-pack sensor)	Seat backrest	HR, HRV	Safety: detection of drunk driving	Laboratory setting	4
7	[32]	2011	Capacitive electrode	Seat backrest	ECG	Sensor development	Laboratary setting, on-road driving	59 and 5
8	[33]	2011	Capacitive electrode, piezoelectric sensor, magnetic impedance sensor	Bucket seat, backrest	ECG, BCG, breath	Sensor development	Static vehicle, on-road driving	1
9	[34]	2011	Capacitive electrode	Seat backrest	ECG	Sensor development	On-road driving	1
10	[35]	2012	Contact electrode	Steering wheel	ECG→HR	Sensor development	On-road driving	8
11	[36]	2012	Contact electrode	Steering wheel	ECG	Sensor development	Laboratory setting	12
12	[37]	2012	Contact electrode	Steering wheel	ECG	Other: driver recognition	Static vehicle	32
13	[38]	2012	Capacitive electrode	Seat backrest	ECG	Sensor development	On-road driving	2
14	[39]	2012	Capacitive electrodes	Bucket seat	ECG	Sensor development	On-road driving	5
15	[40]	2012	Alcohol sensor	Control panel	Alcohol	Sensor development	Driving simulator	1
16	[41]	2013	Contact electrode, capacitive electrode	Steering wheel, bucket seat	ECG	Sensor development	Driving simulator	1
17	[42]	2014	Capacitive electrode (conductive knit fabric)	Seat backrest (cushion)	ECG, EMG	Safety: driving fatigue	Driving simulator	8
18	[18]	2014	Contact electrode (conductive fabric)	Steering wheel	ECG → HRV	Safety: driving fatigue and drowsiness	On-road driving	2
19	[43]	2014	Video camera (eye blinking detector)	Car body (roof handle)	Saccade frequency (eye blinking)	Sensor development	Driving simulator	12
20	[44]	2015	Infrared camera (infrared LEDs)	Windscreen (rear-view mirror)	Video → HR	Sensor development	Laboratory setting	30
21	[25]	2015	Video camera	Windscreen	PPG → HR	Sensor development	On-road driving	10
22	[45]	2015	Radar	Seat backrest	HR, RR	Sensor development	Driving simulator	NA
23	[46]	2015	Video camera	Control panel	Blood volume pulse (BVP) → HR, HRV	Sensor development	Laboratory setting, on-road driving	16 and NA
24	[47]	2015	PPG sensor, pressure sensor, PPG sensors, pressure sensor (gripping), piezoelectric sensor (respiration)	Steering wheel, seat belt	PPG, gripping force, RR	Sensor development	Laboratory setting	NA
25	[48]	2016	Radar	Seat backrest	Heart rate	Sensor development	On-road driving	1
26	[49]	2016	Global Positioning System (GPS)	Car body (OBDII port)	Driving behavior data	Driving behavior profiling	On-road driving	5
27	[50]	2017	Gas sensor (CO2 and alcohol gas sensor), video camera	Steering wheel (steering column, above), windscreen	Gas concentration (CO2 and alcohol), breathing activity	Safety: alcohol detection	On-road driving	10
28	[51]	2017	Magnetic induction sensor	Seat backrest	Respiratory activity	Sensor development	Driving simulator	NA
29	[52]	2017	Spectral photometer, magnetic induction sensor	Safety belt	HR, RR	Sensor development	Laboratory setting	NA
30	[53]	2017	Video camera	Control panel	HR	Sensor development	On-road driving	1
31	[17]	2017	Radar	Steering wheel (under)	HR, RR	Sensor development	On-road driving	5
32	[54]	2017	Radar	Seat, headrest	HR	Sensor development	Laboratory setting	NA
33	[55]	2017	Radar	Seat backrest	HR	Sensor development	On-road driving	8
34	[56]	2018	Infrared camera, pressure pad	Dash board, bucket seat	Body motion	Safety: discomfort detection	Driving simulator	40
35	[57]	2018	BCG sensor (pressure)	Bucket seat (under the foam cushion)	BCG → RR, and HR	Sensor development:existing in-car sensor for new biosignal/information	Laboratory setting	11
36	[58]	2018	Vehicle built-in sensor	Car body (OBD port)	Driving behavior	Diagnosis: mild cognitive impairment	On-road driving	28
37	[59]	2018	IR LED	Steering wheel	PPG → pulse wave velocity	Sensor development	NA	NA
38	[26]	2018	Video camera	Windscreen	HR	Sensor development	On-road driving	10
39	[60]	2018	Radar	Windscreen	HR, RR	Sensor development	Laboratory setting	2
40	[61]	2018	Camera	Control panel	remote PPG (rPPG)	Sensor development	Laboratory setting, on-road driving	12 and 1
41	[62]	2018	Radar	Seat backrest	HR, RR	Sensor development	Laboratory setting	4
42	[63]	2019	Magnetic induction sensor (resonator)	Steering wheel	HR, breathing rate	Sensor development	Static vehicle	2
43	[20]	2019	Capacitive electrode	Seat backrest, bucket seat (seating area)	ECG	Sensor development: sensor performance	Driving simulator	10
44	[64]	2019	Radar	Steering wheel (middle)	HR, RR	Sensor development	Laboratory setting	5
45	[65]	2019	Contact electrode	Steering wheel	HR, RR	Sensor development	Driving simulator	5
46	[11]	2019	Accelerometer	Seat belt	RR	Sensor development	On-road driving	3

**Table 2:** Mapping connections to literature.

Connection	Supporting Literature	Connection	Supporting Literature
S1—L9	[15,18,27,28,29,30,35,36,37,41,65]	S4—B8	[63]
S2—L2	[20,30,33,34,38,39,42]	S4—B21	[33,51,52,63]
S2—L3	[15,20,32,39,41]	S5—B1	[30]
S3—L1	[54,55]	S8—B21	[11]
S3—L2	[45,48,62]	S9—B21	[15,47]
S3—L6	[60]	S9—B31	[47]
S3—L9	[17,64]	S11—B8	[28,31,33,57]
S4—L2	[33,51,63]	S11—B21	[28,57]
S4—L4	[52]	S11—B30	[31]
S5—L9	[30]	S11—B32	[56]
S8—L4	[11]	S13—B8	[25,26,46,53,61]
S9—L4	[15,47]	S13—B23	[43]
S11—L2	[31]	S13—B29	[50]
S11—L3	[28,33,56,57]	S14—B8	[44,61]
S13—L6	[25,26,43,46,50]	S14—B29	[28]
S13—L7	[53,61]	S14—B32	[56]
S14—L6	[44]	S15—B8	[52]
S14—L7	[28,56,61]	S16—B8	[27,29,47]
S15—L4	[52]	S16—B15	[15] [59]
S16—L9	[15,27,29,30,47,59]	S16—B16	[27,30]
S18—L8	[49]	S18—B27	[49]
S19—L8	[58]	S19—B27	[58]
S20—L7	[40]	S20—B28	[40,50]
S20—L9	[50]	B6—P1	[15]
S1—B1	[28]	B8—P2	[18,29,39]
S1—B6	[15,27,30]	B8—P3	[31]
S1—B8	[18,28,29,35,36,65]	B12—P1	[15]
S1—B12	[15,18,29,30,35,37,41,65]	B12—P4	[37]
S1—B21	[65]	B16—P1	[15]
S2—B7	[42]	B21—P1	[15]
S2—B8	[20,39]	B27—P11	[49,58]
S2—B12	[15,20,30,32,33,34,38,39,41,42]	B28—P3	[50]
S3—B8	[17,45,48,54,55,60,62,64]	B30—P3	[31]
S3—B21	[17,45,60,62,64]	B32—P5	[56]

## Abbreviations

The following abbreviations are used in this manuscript:

AI	artificial intelligence
BAN	body area network
BCG	ballistocardiography
CAN	controller area network
cECG	capacitive ECG
ECG	electrocardiography
EDA	electrodermal activity
EMG	electromyography
GPS	Global Positioning System
GSR	galvanic skin response
HIS	health information systems
HR	heart rate
HRV	heart rate variability
IoT	Internet of Things
IVIS	in-vehicle information system
LED	light-emitting diode
MI	magnetic induction
PPG	photoplethysmography
PPGI	PPG imaging
rPPG	remote PPG
RR	respiration rate
SCG	seismocardiography

## Appendix A. Searching String

### Appendix A.1. PubMed

(biosignal[Title] OR biological[Title] OR biological signal[Title] OR biological signals[Title] OR physiological signal[Title] OR physiological parameter[Title] OR physiological parameters[Title] OR vital signal[Title] OR vital signals[Title] OR vital sign[Title] OR vital signs[Title] OR vital parameter[Title] OR vital parameters[Title] OR ECG[Title] OR electrocardiograph[Title] OR electrocardiography[Title] OR electrocardiogram[Title] OR heart rate[Title] OR heart rate variability[Title] OR heartbeats[Title] OR heartbeat[Title] OR respiration rate[Title] OR respiratory rate[Title] OR breathing rate[Title] OR breathing[Title] OR breath[Title] OR respiration[Title] OR body movements[Title] OR body motion[Title] OR driving profile[Title] OR routine[Title])
AND
(car[Title] OR car’s [Title] OR vehicle[Title] OR in-vehicle[Title] OR in-car[Title] OR driver[Title] OR driver’s[Title] OR driving[Title] OR automotive[Title] OR road[Title] OR safety belt[Title] OR steering wheel[Title] OR seat belt[Title])
AND 2009:2019 [edat]

### Appendix A.2. IEEE Xplore

(“Document Title”:biosignal OR “Document Title”:biological OR “Document Title”:biological signal OR “Document Title”:physiological OR “Document Title”:physiological signal OR “Document Title”:physiological parameter OR “Document Title”:vital signal OR “Document Title”:vital sign OR “Document Title”:vital parameter OR “Document Title”:ECG OR “Document Title”:electrocardiograph OR “Document Title”:electrocardiography OR “Document Title”:electrocardiogram OR “Document Title”:heart rate OR “Document Title”:heart rate variability OR “Document Title”:heartbeat OR “Document Title”:respiration rate OR “Document Title”:respiratory rate OR “Document Title”:breathing rate OR “Document Title”:breathing OR “Document Title”:breath OR “Document Title”:respiration OR “Document Title”:body motion OR “Document Title”:driving profile OR “Document Title”:routine OR “Document Title”:driver’s condition OR “Document Title”:health state OR “Document Title”:driver condition)
AND
(“Document Title”:car OR “Document Title”:car’s OR “Document Title”:vehicle OR “Document Title”:in-vehicle OR “Document Title”:in-car OR “Document Title”:driver OR “Document Title”:driver’s OR “Document Title”:driving OR “Document Title”:automotive OR “Document Title”:road OR “Document Title”:safety belt OR “Document Title”:steering wheel OR “Document Title”:seat belt)

### Appendix A.3. Scopus

TITLE (
(“biosignal” OR “biological” OR “biomonitoring” OR “biological signal” OR “physiological signal” OR “physiological parameter” OR “vital signal” OR “vital sign” OR “vital parameter” OR “ECG” OR “electrocardiograph” OR “electrocardiography” OR “electrocardiogram” OR “heart rate” OR “heart rate variability” OR “heartbeat” OR “respiration rate” OR “respiratory rate” OR “breathing rate” OR “breathing” OR “breath” OR “respiration” OR “body movements” OR “body motion” OR “driving profile” OR “routine” )
AND
( “car” OR “car’s ” OR “vehicle” OR “in-vehicle” OR “in-car” OR “driver” OR “driver’s” OR “driving” OR “automotive” OR “road” OR “safety belt” OR “steering wheel” OR “seat belt” )
)
AND
( LIMIT-TO ( PUBYEAR, 2019) OR LIMIT-TO ( PUBYEAR, 2018) OR LIMIT-TO ( PUBYEAR, 2017) OR LIMIT-TO ( PUBYEAR, 2016 ) OR LIMIT-TO ( PUBYEAR, 2015) OR LIMIT-TO ( PUBYEAR, 2014) OR LIMIT-TO ( PUBYEAR, 2013) OR LIMIT-TO ( PUBYEAR, 2012) OR LIMIT-TO ( PUBYEAR, 2011) OR LIMIT-TO ( PUBYEAR, 2010) OR LIMIT-TO ( PUBYEAR, 2009))
AND
( LIMIT-TO ( PUBSTAGE, “final” ))
AND
( LIMIT-TO ( LANGUAGE, “English” ))
